# Supplementary material for: Meta-Quantitative Trait Loci Analysis and Candidate Gene Mining for Drought Tolerance-Associated Traits in Maize (Zea mays L.)
Source: Int J Mol Sci. 2024 Apr 12;25(8):4295. doi: 10.3390/ijms25084295 (PMC11049847; doi:10.3390/ijms25084295)
Supplement: Supplementary file 1 [file ijms-25-04295-s001.zip › Supplementary figures.pdf]

Supplementary Figures

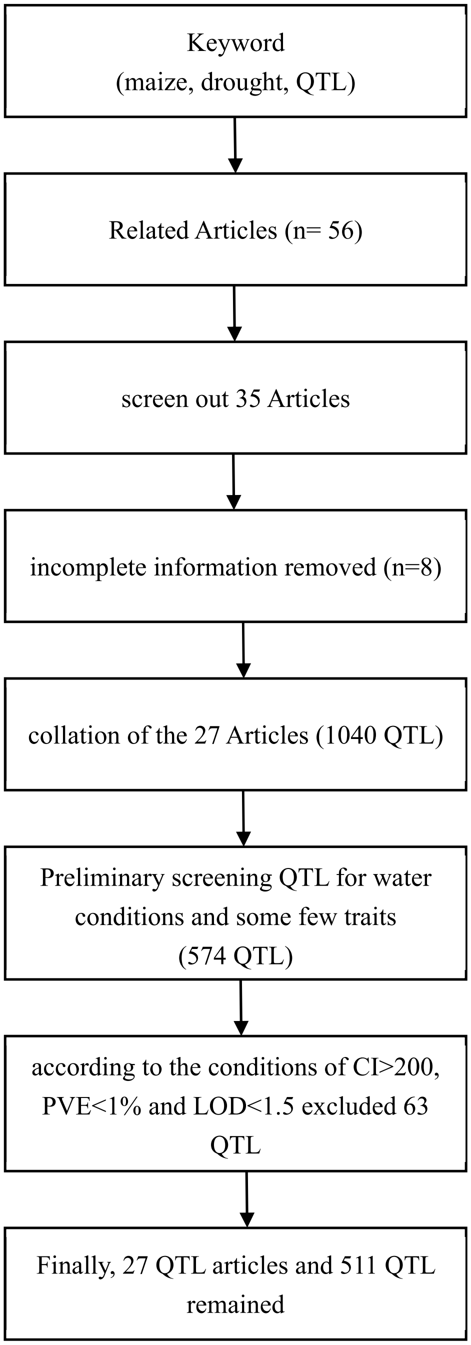

Figure S1. Flow diagram of studies reviewed related to maize drought tolerance

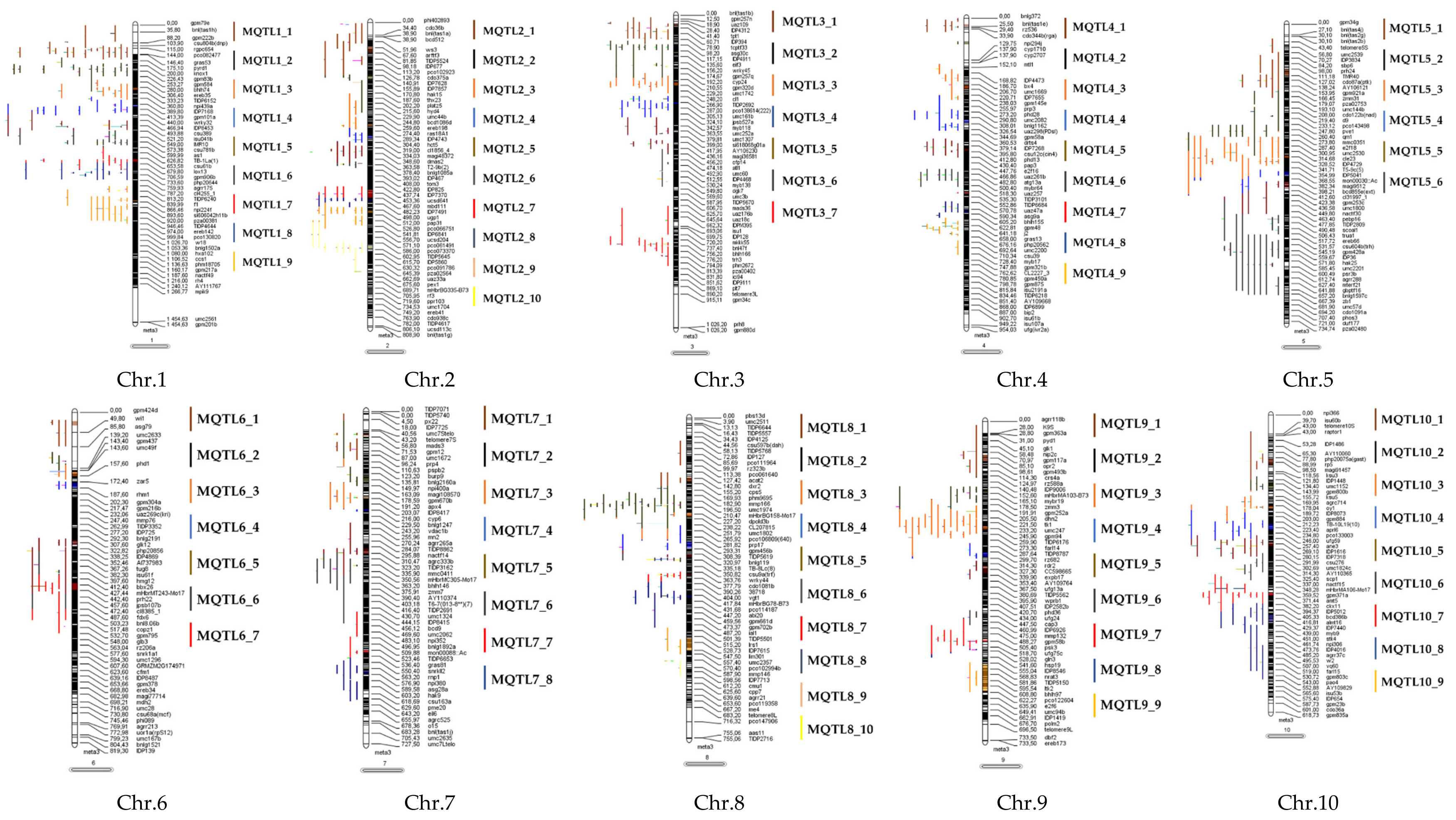
MQTL1_1
MQTL1_2
MQTL1_3
MQTL1_4
MQTL1_5
MQTL1_6
MQTL1_7
MQTL1_8
MQTL1_9
Chr.1
MQTL2_1
MQTL2_2
MQTL2_3
MQTL2_4
MQTL2_5
MQTL2_6
MQTL2_7
MQTL2_8
MQTL2_9
MQTL2_10
Chr.2
MQTL3_1
MQTL3_2
MQTL3_3
MQTL3_4
MQTL3_5
MQTL3_6
MQTL3_7
Chr.3
MQTL4_1
MQTL4_2
MQTL4_3
MQTL4_4
MQTL4_5
MQTL4_6
MQTL4_7
MQTL4_8
MQTL4_9
Chr.4
MQTL5_1
MQTL5_2
MQTL5_3
MQTL5_4
MQTL5_5
MQTL5_6
Chr.5
MQTL6_1
MQTL6_2
MQTL6_3
MQTL6_4
MQTL6_5
MQTL6_6
MQTL6_7
Chr.6
MQTL7_1
MQTL7_2
MQTL7_3
MQTL7_4
MQTL7_5
MQTL7_6
MQTL7_7
MQTL7_8
Chr.7
MQTL8_1
MQTL8_2
MQTL8_3
MQTL8_4
MQTL8_5
MQTL8_6
MQTL8_7
MQTL8_8
MQTL8_9
MQTL8_10
Chr.8
MQTL9_1
MQTL9_2
MQTL9_3
MQTL9_4
MQTL9_5
MQTL9_6
MQTL9_7
MQTL9_8
MQTL9_9
Chr.9
MQTL10_1
MQTL10_2
MQTL10_3
MQTL10_4
MQTL10_5
MQTL10_6
MQTL10_7
MQTL10_8
MQTL10_9
Chr.10

Figure S2. Distribution of MQTL on all the 10 maize chromosomes. Vertical lines on the left of chromosomes indicate the CI of the initial QTL, horizontal lines indicate the variance. The initial QTL with the same color of each chromosome is aggregated into an MQTL, MQTL is showing in different color right beside each chromosome, genetic distance (cM) and the marker of IBM2 2008 neighbors on the right of chromosome.
